# Analysis of the Feasibility of Concurrent Application of Magnetic Nanoparticles as MRI Contrast Agents and for Magnetic Hyperthermia

**DOI:** 10.3390/jfb17010054

**Published:** 2026-01-21

**Authors:** Przemysław Wróblewski, Michał Wieteska, Mateusz Midura, Grzegorz Domański, Damian Wanta, Wojciech Obrębski, Tomasz Płociński, Ewa Piątkowska-Janko, Kamil Lipiński, Mikhail Ivanenko, Mateusz Orzechowski, Waldemar T. Smolik, Piotr Bogorodzki

**Affiliations:** 1 Institute of Radioelectronics and Multimedia Technology, The Faculty of Electronics and Information Technology, Warsaw University of Technology, Nowowiejska 15/19, 00-655 Warsaw, Poland; przemyslaw.wroblewski@pw.edu.pl (P.W.); michal.wieteska@pw.edu.pl (M.W.); mateusz.midura@pw.edu.pl (M.M.); grzegorz.domanski@pw.edu.pl (G.D.); wojciech.obrebski@pw.edu.pl (W.O.); kamil.lipinski.dokt@pw.edu.pl (K.L.); mikhail.ivanenko.dokt@pw.edu.pl (M.I.); mateusz.orzechowski@pw.edu.pl (M.O.); waldemar.smolik@pw.edu.pl (W.T.S.); piotr.bogorodzki@pw.edu.pl (P.B.); 2 Faculty of Materials Science and Engineering, Warsaw University of Technology, Wołoska 141, 02-507 Warsaw, Poland; tomasz.plocinski@pw.edu.pl; 3 Nalecz Institute of Biocybernetics and Biomedical Engineering PAN, Księcia Trojdena 4, 02-109 Warsaw, Poland; ewa.piatkowska-janko@pw.edu.pl

**Keywords:** Nuclear Magnetic Resonance, relaxation, calorimetry, magnetic nanoparticles, magnetic hyperthermia, diffusion

## Abstract

The aim of the article was to analyze the potential simultaneous use of magnetic nanoparticles as contrast agents in MRI imaging and for magnetic hyperthermia. The study proposed characterizing the nanoparticles using various measurement methods in order to investigate the relationships between different properties. The first stage involved measuring images of nanoparticle samples using scanning transmission electron microscopy (TEM) and dynamic light scattering (DLS). The diameter distribution of nanoparticles was determined based on image segmentation. The next step involved measuring relaxation properties of nanoparticles in low and high magnetic fields. The research was carried out for nanoparticle solutions of various concentrations and properties. The last step was measuring calorimetric properties of nanoparticles as a thermal source under alternating magnetic field excitation conditions. The range of nanoparticle diameters (20–25 nm) for which maximum losses occur in an alternating magnetic field corresponds to the diameter range in which the maximum r_2_ relaxivity is observed.

## 1. Introduction

Magnetic nanoparticles have great application possibilities in medicine, in particular in hyperthermia and imaging diagnostics [1,2,3,4,5,6,7]. Magnetic hyperthermia is a therapeutic technique that uses magnetic nanoparticles to generate heat for medical treatment, most commonly in cancer therapy [8,9,10,11]. In this method, magnetic nanoparticles are introduced into a tumor and then exposed to an alternating magnetic field. The particles respond to the field by producing heat, which raises the local temperature (typically to 41–45 °C). This controlled heating can damage or kill cancer cells while minimizing harm to surrounding healthy tissue, and it is often used in combination with chemotherapy or radiotherapy [12,13,14,15]. Superparamagnetic iron oxide nanoparticles can be chemically synthesized using various methods [16]. In the case of hyperthermia, the aim is to maximize the thermal losses generated in the tumor volume while minimizing the power of losses induced by eddy currents in the patient’s body. The heating efficiency of magnetic nanoparticles in magnetic hyperthermia is commonly quantified by the specific absorption rate (SAR), defined as the power absorbed per unit mass of magnetic material [17,18]. The SAR is typically expressed in watts per gram (W g^−1^) and represents the ability of nanoparticles to convert electromagnetic energy into heat in an alternating magnetic field. The SAR value depends on several factors, including the intrinsic properties of the nanoparticles (such as size, composition, magnetic anisotropy, and saturation magnetization), as well as external parameters like the amplitude and frequency of the applied magnetic field and the surrounding medium. The therapeutic effectiveness of hyperthermia is based on the principle that cancer cells are more vulnerable to heat due to their elevated metabolic activity [19]. However, in diagnostics, one of the most important problems is the elimination of magnetic losses that cause an undesirable increase in temperature during imaging.

Our current research studies involve modeling the applications of nanoparticles and developing measurement methods designed for specific applications. Nanoscale magnetic particles possess unique physical and particularly magnetic properties extremely different from those of their bulk counterparts [20]. The power of magnetic losses, i.e., the heating capacity of nanoparticles, depends on their hydrodynamic diameter. Measuring the hydrodynamic diameter is an extremely difficult task, and its value is not provided by manufacturers. The core diameter of nanoparticles provided is a rough estimate and does not account for the dispersion of particle geometry. This work presents combined nanoparticle characterization methods that allow for the determination of some nanoparticle parameters, including dynamic magnetic power losses, relaxation constants, diffusion coefficient, and hydrodynamic diameter. Biocompatibility polymer-coated IONPs (iron oxide nanoparticles) have been found to be relatively nontoxic and utilized to improve the biocompatibility of IONPs [21]. The methods used to determine diameter distribution of nanoparticles are scanning electron microscopy (SEM), transmission electron microscopy (TEM) and dynamic light scattering (DLS). There are two primary electron imaging techniques: conventional transmission electron microscopy (TEM) and scanning transmission electron microscopy (STEM). In TEM, a parallel electron beam illuminates a wide area of the sample. The transmitted beam is then processed by the objective lens, which, depending on its settings, produces either a diffraction pattern or an image that reveals valuable crystallographic details. In contrast, STEM employs a focused, convergent beam that targets a small region of the sample and scans across its surface. At each scanning point, the beam passes through the sample, and various detectors are used to collect different types of data [22]. In this study, only the STEM technique was employed. Another technique, scanning electron microscopy (SEM), utilizes a focused beam of electrons to scan the surface of the sample, enabling high-resolution imaging; however, SEM was not used in this work [23]. DLS is a widely utilized method in scientific and industrial sectors where the distribution of particle size is crucial [24,25]. This method utilizes Brownian motion of particles in suspension to measure their size. The random motion of nanoparticles in a liquid depends on the particle size. Small particles move quickly, and large particles move slowly.

Magnetic resonance imaging (MRI) is widely used for disease diagnosis due to its excellent soft tissue contrast and non-ionizing nature. The diagnostic performance of MRI relies on sufficient signal contrast between lesions and healthy tissue; therefore, contrast agents are often required, particularly for early-stage disease detection [21]. However, to improve sensitivity and distinguish between healthy and diseased tissue, particularly in the early stages, contrast agents (CAs) are often necessary. These agents enhance image contrast by reducing the longitudinal (T_1_) and transverse (T_2_) relaxation times of water protons, leading to improved image clarity and enabling more effective non-invasive visualization, ultimately supporting better clinical outcomes and patient care [26,27]. Although superparamagnetic iron oxide nanoparticles are widely regarded as biocompatible contrast agents, recent studies indicate that, like other metal oxide nanoparticles, they may exhibit a certain degree of toxicity under specific conditions. Experimental investigations using both in vitro and in vivo models have demonstrated that exposure to metal oxide nanoparticles can lead to variable toxic effects, depending on their physicochemical characteristics. Nevertheless, compared with oxides of other metals, iron oxide nanoparticles remain among the most favorable nanomaterials for biomedical applications due to their relatively low toxicity, superparamagnetic behavior, high stability in aqueous environments, and good biocompatibility. Importantly, the biological impact of magnetic nanoparticles is highly dependent on multiple factors, including particle size, shape, structure, surface functionalization, concentration, administered dose, biodistribution, bioavailability, solubility, immunogenicity, and pharmacokinetic profile [28,29,30,31,32]. One of the problems associated with superparamagnetic particles is that the negative contrast produced by these nanoparticles is also generated, for example, by air bubbles or calcifications [33]. In this work we tested whether proton relaxation times and diffusion coefficient obtained in the magnetic resonance imaging can be used to determine the hydrodynamic volume of nanoparticles. The tests were performed for nanoparticle solutions of various concentrations and properties.

The measurement methods presented in this paper allow for the assessment of the hydrodynamic diameter of nanoparticles and thus for the assessment of the heating of nanoparticles when stimulated with an alternating magnetic field. For this purpose, the system described earlier [34] was adopted. The values of losses measured in the system were compared with the temperature increase measured calorimetrically using a fiber-optic thermometer.

## 2. Materials and Methods

### 2.1. Heat Generation Theory

**Figure 1:**
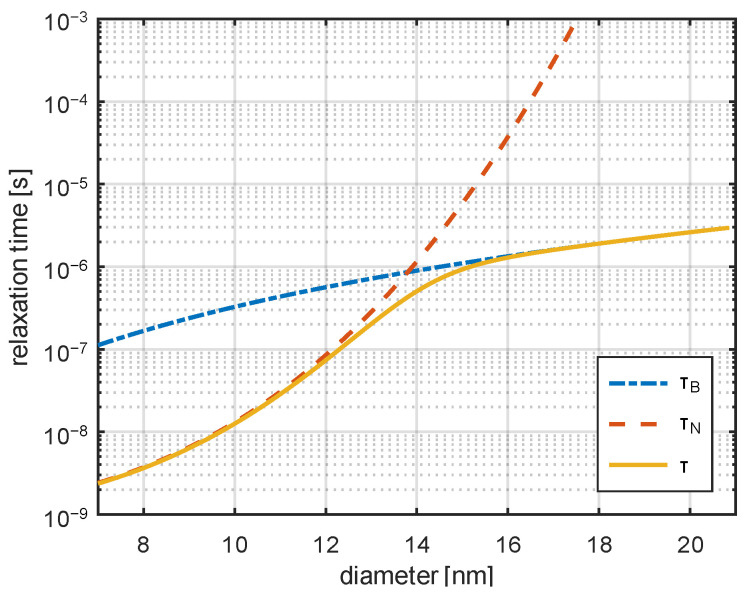
Relaxation times calculated for magnetite nanoparticles dispersed in water K = 21 kJ/m^3^, T = 310 K, η = 8.9 × 10^−4^ Pa·s) according to Equations (1)–(3) [35,36,37]. The effective relaxation time is marked with a dashed line.

Heat generation in magnetic nanoparticles arises from two relaxation mechanisms: Néel and Brownian relaxation. The relaxation time characterizes the response of nanoparticles to an alternating magnetic field. In Néel relaxation, heat is generated through the reorientation of the magnetic moment within the nanoparticle without physical rotation, whereas Brownian relaxation involves the physical rotation of the entire nanoparticle within the surrounding fluid (Figure 1).

The Néel relaxation time $\tau_{N}$ depends on the magnetic properties of the nanoparticle and is given by [35]

(1) $$\tau_{N}=\tau_{0}exp(KV/k_{B}T),$$

where *K*—effective anisotropy constant, *V*—volume of the particle, *k_B_*—Boltzmann constant (*k_B_* = 1.38 × 10^−23^ J/K), *T*—temperature on the absolute scale, and *τ*_0_ ≈ 10^−9^ s.

The second mechanism responsible for heat generation is the Brownian process with a relaxation time constant equal to [36]

(2) $$\tau_{B}=\frac{3V_{h}\eta }{k_{B}T},$$

where V_h_—hydrodynamic volume of the particle [m^3^], and η—viscosity of the liquid [Pa·s] in which the nanoparticles are immersed. It involves the fluctuation of the particle’s orientation under the influence of an external magnetic field.

The effective relaxation time for a particle in which both processes take place is given by [37]

(3) $$\tau =\frac{\tau_{N}\tau_{B}}{\tau_{N}+\tau_{B}}.$$

An example of the dependence of relaxation times on the diameter of nanoparticles is shown in Figure 1. The magnetization M is given by [38,39]

(4) $$M=\frac{{N_{m}M}_{SP}\pi d_{m}^{3}}{6}\cdot \left[\coth\left(\xi \right)-\frac{1}{\xi }\right],$$

where the argument ξ is given by the formula [38,39]

(5) $$\xi =\frac{\mu_{0}HM_{SP}\pi d_{m}^{3}}{{6k}_{B}T}.$$

In the above formulas, H means the magnetic field strength [A/m], M_SP_—saturation magnetization of the nanoparticle core material, μ_0_—vacuum magnetic permeability, μ_0_ = 1.257 × 10^−6^ H/m, nanoparticles have a diameter of d_m_, and N_m_—number of magnetic moments in the unit volume (number of nanoparticles). The saturation magnetization of the sample M_s_ with nanoparticles is equal [38,39]

(6) $$M_{s}=N_{m}\frac{M_{SP}\pi d_{m}^{3}}{6}.$$

In the linear response regime [40], magnetization can be expressed using magnetic susceptibility χ as

(7) M=χH.

In response to excitation by an external magnetic field with circular frequency ω = 2πf, where f—frequency, and amplitude H_0_ is given by the formula [40]

(8) $$H=H_{0}\cos\left(\omega t\right)$$

the resulting magnetization is given by the formula [35]

(9) $$M\left(t\right)=H_{0}\left(\chi^{\prime }\cos\left(\omega t\right)+\chi^{\prime \prime }\sin\left(\omega t\right)\right),$$

where χ″—imaginary part of magnetic susceptibility, given by the formula [35]

(10) $$\chi^{\prime \prime }=\frac{\tau \omega }{1+{\left(\tau \omega \right)}^{2}}\chi_{0}$$

The complex magnetic susceptibility is given by the formula [40]

(11) $$\chi =\chi^{\prime }-j\chi^{\prime \prime }$$

The parameter χ_0_, called equilibrium magnetic susceptibility, is given by the formula [41]

(12) $$\chi_{0}=\frac{\mu_{0}M_{s}^{2}V}{3k_{B}T},$$

The real part of the magnetic susceptibility can be calculated from the formula [40]

(13) $$\chi^{\prime }=\frac{1}{1+{\left(\tau \omega \right)}^{2}}\chi_{0}.$$

**Figure 2:**
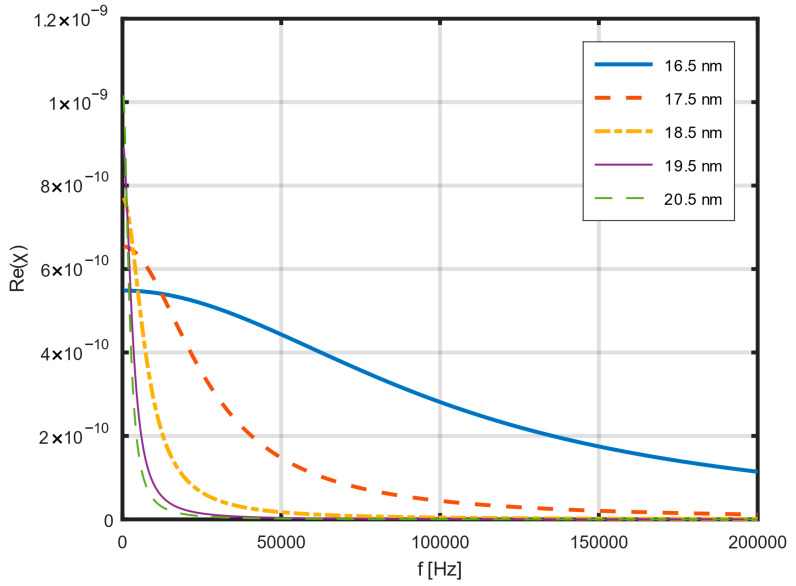
Dependence of the real part of magnetic susceptibility on frequency for several nanoparticle diameters in the range up to 200 kHz, calculated using Equations (10)–(13) [35,40,41].

**Figure 3:**
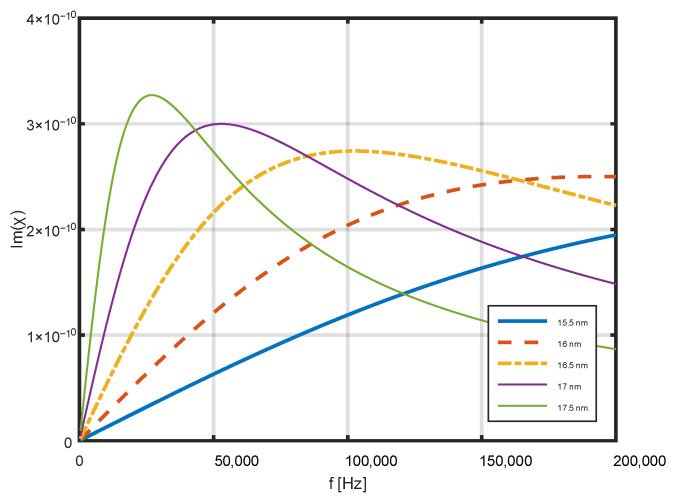
Dependence of the imaginary part of magnetic susceptibility on frequency for several nanoparticle diameters in the range up to 200 kHz, calculated using Equations (10)–(13) [35,40,41].

Figure 2 shows an example of the dependence of the actual magnetic susceptibility on frequency. Calculations were performed for several nanoparticle diameters: from 16 nm to 20 nm. The ratio of the hydrodynamic diameter to the nanoparticle diameter was 1.5. In the selected range of nanoparticle diameters, the real component of magnetic permeability strongly depends on both the field frequency and the size of the nanoparticles. Figure 3 shows an example of the dependence of the imaginary component of magnetic susceptibility on frequency in the range from 0 to 200 kHz for several nanoparticle diameters.

There is a very strong dependence of the imaginary component of the magnetic susceptibility of nanoparticles on their diameter. Therefore, selecting the right diameter is a key issue to ensure the good heating efficiency of such nanoparticles.

Heating power is proportional to imaginary part of magnetic susceptibility of nanoparticles [42,43]

(14) $$P=f\Delta U=f\pi \mu_{0}H_{0}^{2}\chi^{\prime \prime }$$

The energy released in one cycle is given by [44]

(15) $$\Delta U=-\mu_{0}\oint MdH$$

The above integral gives the hysteresis loop area. This area is proportional to the saturation magnetization of the core material and then to the magnetic moment of nanoparticles.

### 2.2. Relaxation Rate Theory

Another application of magnetic nanoparticles in medicine is enhancing contrast in magnetic resonance imaging (MRI). An NMR (Nuclear Magnetic Resonance) measurement is performed by initially polarizing an ensemble of nuclear moments, then observing their relaxation in time [45]. The inhomogeneous field may be due to intrinsic structures, such as iron-rich cells in the brain and deoxygenated red blood cells, or it may be induced by administering a contrast agent [46]. Magnetic nanoparticles locally alter the magnetic field strength, affecting the relaxation processes of hydrogen nuclei in water molecules, which serve as the natural medium in which the nanoparticles form a suspension. Relaxation processes can be divided into two types: spin-lattice relaxation, which means the return of the population of spins to their Boltzmann distribution, and spin–spin, in which coherence decays. Let us look at the one-dimensional theory first. Spin–grid relaxation is also called longitudinal relaxation, and spin–spin is also called transverse relaxation. Let us consider a set of uncoupled 1/2 spins exposed to two magnetic forces: a strong static field B_0_ along the z-axis (longitudinal) and a weak field B_x_(t) along the x-axis (transverse). Fluctuating cross fields have a zero mean value and a non-zero mean value of the square of the field. Susceptibility-induced T_2_ shortening is caused by the dephasing of magnetic moments due to magnetic field gradients produced by small magnetized particles [47]. Proton T_2_ relaxation in the presence of iron oxide nanoparticles can be described by dipole–dipole coupling, Curie spin relaxation, and scalar relaxation mechanisms [21]. According to the outer-sphere theory, proton dephasing occurs in three regimes: the Motional Averaging Regime (MAR), the Static Dephasing Regime (SDR), and the Echo-Limiting Regime (ELR) [20,21,48]. As nanoparticle size increases, T_2_ relaxivity (r_2_) initially rises in the MAR, reaches a maximum in the SDR, and subsequently decreases in the ELR due to restricted proton diffusion. These size-dependent transitions indicate the existence of an optimal nanoparticle diameter for maximizing transverse relaxivity. The contrast-enhancing efficiency is proportional to magnetic particle concentration $C_{Fe}$, and described using relaxivity coefficient r_1_ and r_2_ [49]

(16) $$\frac{1}{T_{i}}=\frac{1}{T_{i,water}}+r_{i}C_{Fe},\;\;i=1,2.$$

The total relaxivity of the is the sum of the water relaxivity (host liquid) and relaxivity resulting from the non-zero concentration of the contrast agent. There are two mechanisms of nuclear relaxation. The first one is based on thermally induced fluctuations of the magnetic moment of paramagnetic particles. The second one is related to motion of the host molecules through a steady-state magnetic field that is non-uniform in space due to magnetic nanoparticles. This mechanism is presented in liquid systems only and is determined by the self-diffusion of the host molecules. The characteristic time of this process is given by [50]

(17) $$\tau_{D}=\frac{R^{2}}{D},$$

where R is radius of the nanoparticle and D is the diffusion constant. The relaxation rates depend on two spectral density functions. The first spectral density function related to proton diffusion in a nonuniform magnetic field is given by [50]

(18) $$J^{A}(z)=\frac{1+\frac{5z}{8}+\frac{z^{2}}{8}}{1+z+\frac{z^{2}}{2}+\frac{z^{3}}{6}+\frac{{4z}^{4}}{81}+\frac{z^{5}}{81}+\frac{z^{6}}{648}},$$

The second spectral density function accounting both for proton diffusion and for the fluctuation of the magnetic moment around its mean value is given by [50]

(19) $$J^{F}(\omega ,\tau_{D},\tau_{N})=Re\left[\frac{1+\frac{\Omega^{1/2}}{4}}{1+\Omega^{1/2}+4\Omega /9+\Omega^{\frac{3}{2}}/9}\right],$$

where

(20) $$\Omega =\left(i\omega +\frac{1}{\tau_{N}}\right)\tau_{D},$$

The relaxation rates for the case large magnetic crystals are given by [50]

(21) $$\frac{1}{T_{1}}=\frac{32\pi \hbar^{2}\gamma_{I}^{2}\gamma_{S}^{2}N_{A}C}{135000\cdot RD}\left[3J^{F}\left(\omega_{I},\tau_{D},\tau_{N}\right)∆S_{z}^{2}+3J^{A}\left(\sqrt{2\omega_{I}\tau_{D}}\right){\langle S_{z}\rangle }^{2}\right],$$

and

(22) $$\frac{1}{T_{2}}=\frac{32\pi \hbar^{2}\gamma_{I}^{2}\gamma_{S}^{2}N_{A}C}{135000\cdot RD}\left[\left(\frac{3}{2}J^{F}\left(\omega_{I},\tau_{D},\tau_{N}\right)+2J^{F}\left(0,\tau_{D},\tau_{N}\right)\right)∆S_{z}^{2}+\;\left(\frac{3}{2}J^{A}\left(\sqrt{2\omega_{I}\tau_{D}}\right)+2J^{A}\left(0\right)\right){\langle S_{z}\rangle }^{2}\right],$$

where C is nanoparticle concentration in moles/liter, $N_{A}\;$is the Avogadro number, $\gamma_{I}$ is the proton gyromagnetic constant, $\gamma_{S}$ is the electron gyromagnetic constant, and $S_{Z}$ is the spin projection on the z-axis. The relation between variance and mean values of the spin projection on the z-axis is given by relaxation rates are given by [50]

(23) $$∆S_{z}^{2}={\langle S_{Z}^{2}\rangle -\langle S_{z}\rangle }^{2}$$

The microscopic outer sphere theory leads to another expressions for relaxation rates [51]

(24) $$\frac{1}{T_{1}}=\frac{32\pi \hbar^{2}\gamma_{I}^{2}\gamma_{S}^{2}{S(S+1)N}_{A}C}{405000\cdot RD}\left[7J\left({\tau_{D}\omega }_{S}\right)+3J\left({\tau_{D}\omega }_{I}\right)\;\right],$$

and

(25) $$\frac{1}{T_{2}}=\frac{32\pi \hbar^{2}\gamma_{I}^{2}\gamma_{S}^{2}{S(S+1)N}_{A}C}{405000\cdot RD}\left[6.5J\left({\tau_{D}\omega }_{S}\right)+1.5J\left({\tau_{D}\omega }_{I}\right)+2J(0)\right],$$

where the function J is given by [51]

(26) $$J(\omega )=Re\left[\frac{1+\frac{{\left(i\omega \tau_{D}\right)}^{1/2}}{4}}{1+{\left(i\omega \tau_{D}\right)}^{1/2}+4(i\omega \tau_{D})/9+{\left(i\omega \tau_{D}\right)}^{\frac{3}{2}}/9}\right].$$

The square of the magnetic moment of each particle $\mu^{2}$ is given by [51]

(27) $$\mu^{2}=\gamma_{S}^{2}\hbar^{2}S(S+1)$$

After replacement one can get the final form of the equations for relaxation rates [51]:

(28) $$\frac{1}{T_{1}}=\frac{32\pi \gamma_{I}^{2}{\mu^{2}N}_{A}C}{405000\cdot RD}\left[7J\left({\tau_{D}\omega }_{S}\right)+3J\left({\tau_{D}\omega }_{I}\right)\;\right],$$

and

(29) $$\frac{1}{T_{2}}=\frac{32\pi \hbar^{2}\gamma_{I}^{2}{\mu^{2}N}_{A}C}{405000\cdot RD}\left[6.5J\left({\tau_{D}\omega }_{S}\right)+1.5J\left({\tau_{D}\omega }_{I}\right)+2J(0)\right].$$

The relaxation rates depend on the square of the magnetic moment of nanoparticles.

### 2.3. Magnetic Nanoparticles

The group of nanoparticles used in the measurements came from Ocean NanoTech and were labeled as follows: SPA30-10 (30 nm), SPA25-10 (25 nm), SPA20-10 (20 nm), SPA15-10 (15 nm), and SPA10-10 (10 nm) in diameter [52]. These water-soluble iron oxide nanoparticles are superparamagnetic particles characterized by excellent colloidal stability and a narrow size distribution. Synthesized via a thermal decomposition method, the nanoparticles consist of single-crystalline iron oxide cores, which according to the manufacturer may exhibit either a magnetite (Fe_3_O_4_) or maghemite (γ-Fe_2_O_3_) structure. As no additional phase-specific structural characterization was performed in this study, the nanoparticles are referred to hereafter using the general term “iron oxide nanoparticles”.

Utilizing a proprietary monolayer polymer coating technology, the manufacturer transformed hydrophobic, organic ligand-coated iron oxide nanoparticles into water-dispersible forms. These nanoparticles are engineered for exceptional stability under demanding conditions.

Key features of the examined nanoparticles include uniform particle size distribution, superior colloidal stability, and easy purification using a proprietary magnetic column system. The nanoparticles were supplied in water with a concentration of 5 mg/mL and contained no additional reactive surface groups.

### 2.4. Measurement Methods

In order to compare the dependence of the heating efficiency of nanoparticle suspensions on their other parameters, e.g., size, relaxation times and magnetic susceptibility, temperature increases during suspensions exposed to an alternating magnetic field were measured. The efficiency of heating the medium by the magnetic field can be described by the Specific Absorption Rate (SAR). The SAR can be calculated using the formula [53]

(30) $$SAR=\frac{C\rho }{c_{n}}\left(\frac{dT}{dt}\right){|}_{t=0},$$

where t—time, C—specific heat of the suspension, ρ—density of the suspension, c_n_—concentration of nanoparticles in the suspension, ${\left.\frac{dT}{dt}\right|}_{t=0}$—initial value of the slope of the temperature increase curve over time.

The next step was to measure the hydrodynamic diameters using the dynamic laser light scattering method. This technique enables the measurement of the so-called hydrodynamic diameter of nanoparticles, which is larger than the diameter of the nanoparticle itself, considered as the sum of the core diameter and twice the thickness of the polymer coating. The measurements were carried out using a Zetasizer Nanoseries ZS (Malvern Instruments) device, equipped with a He–Ne laser (4 mW) operating at 632.8 nm.

Dependance of T_1_ and T_2_ relaxation times on nanoparticles concentration was measured in an Oxford Instruments MQC benchtop Nuclear Magnetic Resonance analyzer. The value of the magnetic field induction was 0.55 T. The relaxation times were converted into relaxation constants. Dependance of T_1_ and T_2_ relaxation times on particles diameter was measured in a Bruker scanner for nanoparticles with diameters of 10 nm, 15 nm, 20 nm, 25 nm, and 30 nm. The value of the magnetic field induction was 7 T. The relaxation times were also converted into relaxation constants.

**Figure 4:**
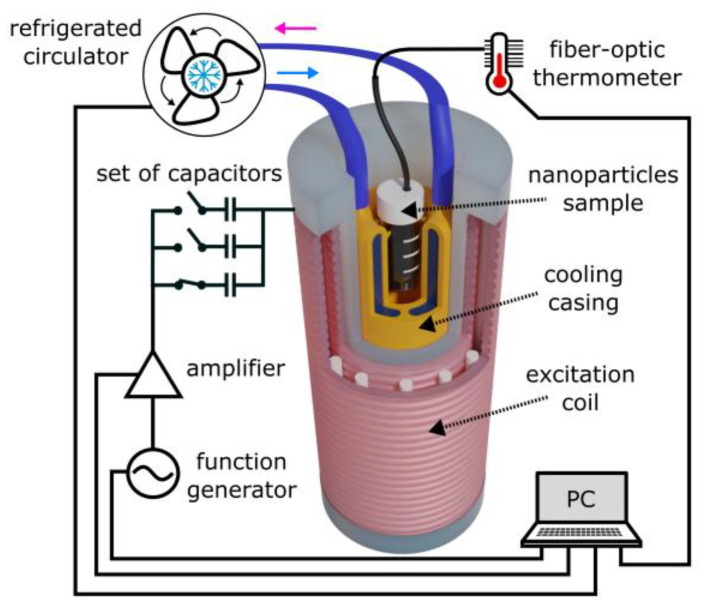
Block diagram of the developed system, described in [20], in the configuration for measuring the heating efficiency of nanoparticles.

A setup was built to measure the properties of nanoparticles [34]. The block diagram of the measurement system in the configuration for measuring the heating efficiency of nanoparticles is shown in Figure 4. This setup was used to measure both the magnetic permeability and magnetic loss of nanoparticles. In order to carry out magnetic permeability measurements, the system was equipped with excitation and detection parts. The excitation part included an Agilent 33522A function generator used to produce sinusoidal excitation signal, which was then amplified by an AE Techron 7228 power amplifier and was fed to a resonant circuit consisting of a set of capacitors and a litz-wire transmitting coil. The entire measurement process control and processing of recorded signals was performed in a program created in the MATLAB R2022b environment. Alternatively, the excitation part was used in calorimetric measurements of magnetic losses of nanoparticles as a source of an alternating magnetic field to heat the nanoparticles.

In this case, the temperature increase of the nanoparticle suspension was recorded using a fiber-optic thermometer. The ability to study the magnetic parameters of nanoparticles and measure their heating efficiency within the same measurement geometry has simplified the methodology for investigating the properties of nanoparticles.

## 3. Results

The nanoparticles were characterized using various measurement methods in order to determine the relationships between different properties.

### 3.1. Measurement of Nanoparticle Geometrical Properties 

**Figure 5:**
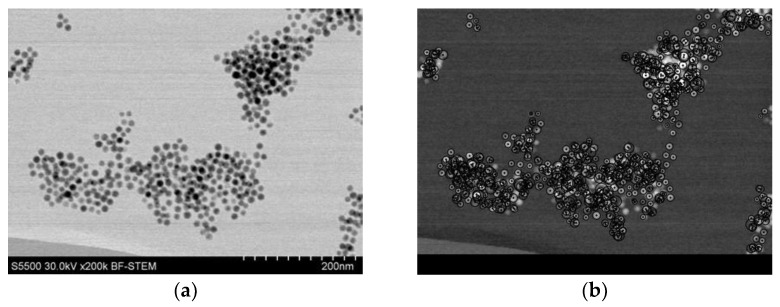
Side-by-side comparison of nanoparticle images: (**a**) original STEM image of iron oxide nanoparticles with a nominal diameter of 10 nm and (**b**) final processed image after grayscale inversion, filtering, segmentation, and watershed separation used for automated diameter estimation.

**Figure 6:**
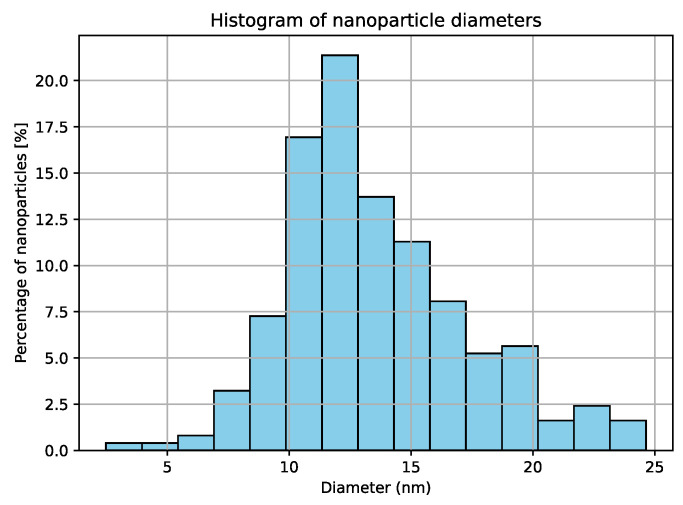
Distribution of 10 nm nanoparticle diameters measured by TEM method.

An image of nanoparticles obtained using a Hitachi STEM S5500 electron microscope is shown in Figure 5. STEM allows for the measurement of core diameter distributions of nanoparticles and thus enables verification of the data provided by the manufacturer. The estimation of diameter distributions was carried out using computer image analysis performed with custom-written software developed by one of the authors in the Python 3.8 programming language. The diameter distribution is shown in Figure 6. The obtained image has good contrast, and the diameter of the nanoparticle cores corresponds to the diameter range declared by the manufacturer. The original STEM image was subjected to grayscale inversion, resulting in a negative image in which nanoparticles appeared as bright objects on a dark background. A median filter was then applied to reduce noise while preserving object edges. Subsequently, thresholding using Otsu’s method [54] was employed to automatically separate nanoparticles from the background. Morphological closing was applied to fill small holes and improve particle connectivity. A distance transform was then used to identify particle centers, followed by watershed segmentation to separate adjacent nanoparticles. This step enabled accurate counting and measurement of particles located in close proximity. For each segmented object, a minimum enclosing circle was determined, and its diameter was used as an estimate of the nanoparticle core diameter.

The corresponding diameter distribution obtained from image analysis is shown on Figure 6. The peak of the diameter distribution occurs at approximately 12 nm, which is consistent with the nominal diameter of 10 nm provided by the manufacturer. The relatively long tail of the distribution extending toward larger diameters is attributed to strong nanoparticle aggregation and the limitations of automatic segmentation for closely packed particles in STEM images.

### 3.2. Hydrodymamical Diameter Measurement of Nanoparticles

**Figure 7:**
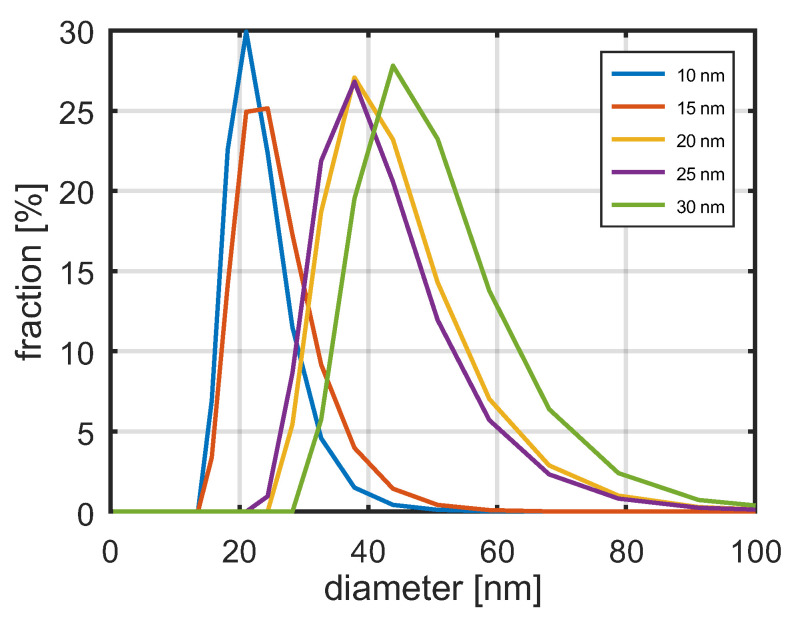
Distribution of hydrodynamic diameters of nanoparticles measured by DLS method.

The distribution of hydrodynamic diameters of SPA nanoparticles, measured using the DLS technique, is shown in Figure 7. The graph shows the distributions of hydrodynamic diameters measured for SPA magnetic nanoparticles with nominal diameters ranging from 10 nm to 30 nm. An increase in hydrodynamic diameter is observed with increasing core diameter of the nanoparticles, ensuring their good heating efficiency.

The determined hydrodynamic diameters are significantly larger than the nanoparticle diameters measured using TEM. The particle size measured by the DLS technique is often overestimated in comparison to the TEM method due to adsorbing layer on the particle surface [55].

As the core diameters of the nanoparticles increase, the variance of their hydrodynamic diameters also increases. The distributions of hydrodynamic diameters exhibit long tails toward larger values. This may be due to the effect of nanoparticle aggregation as they can form clusters that correspond to nanoparticles with larger diameters.

### 3.3. Measurement of Nanoparticle Relaxation Properties 

**Figure 8:**
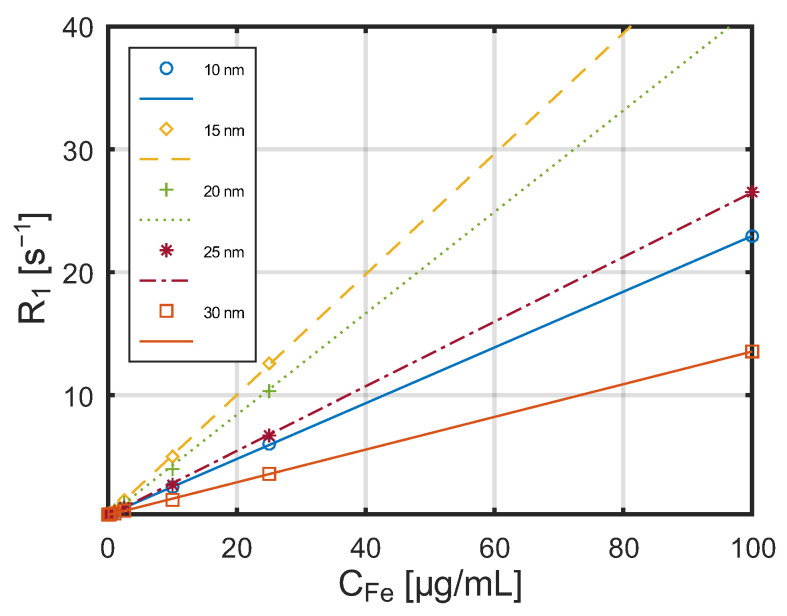
Dependence of the relaxation constant R_1_ on the concentration of SPA nanoparticles measured by the MQC benchtop NMR spectrometer) in a 0.55 T magnetic field.

A series of studies was conducted on the influence of nanoparticles with different diameters on T_1_ and T_2_ relaxation times at low and high magnetic field strengths. The measured dependence of the relaxation constant R_1_ on SPA nanoparticle concentration, obtained using the MQC benchtop NMR spectrometer at a magnetic field strength of 0.55 T, is shown in Figure 8.

**Figure 9:**
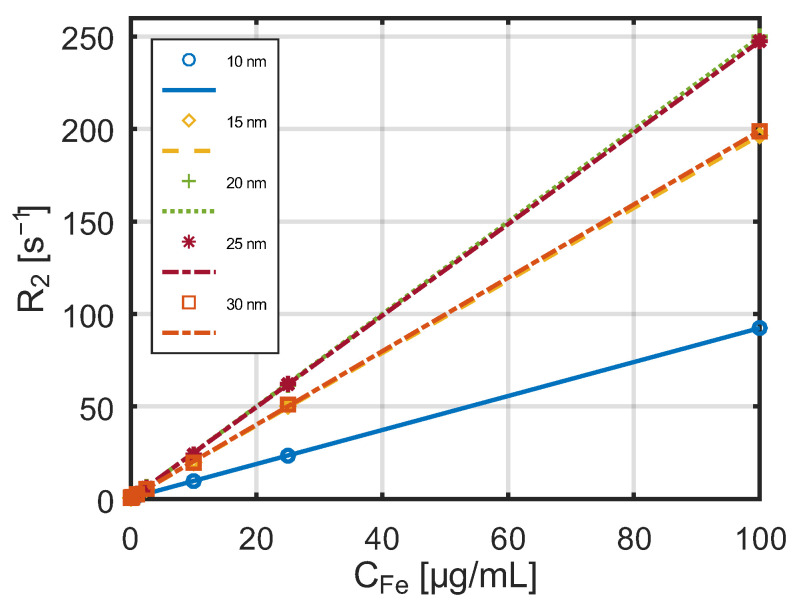
Dependence of the relaxation constant R_2_ on the concentration of SPA nanoparticles measured by the MQC benchtop NMR spectrometer in a 0.55 T magnetic field.

The dependence of the relaxation constant R_2_ on SPA nanoparticle concentration at the same magnetic field strength (0.55 T) is presented in Figure 9.

In both cases, a linear relationship is observed between the reciprocals of the relaxation times (relaxation rates) and the concentration of nanoparticles in the aqueous suspensions.

**Figure 10:**
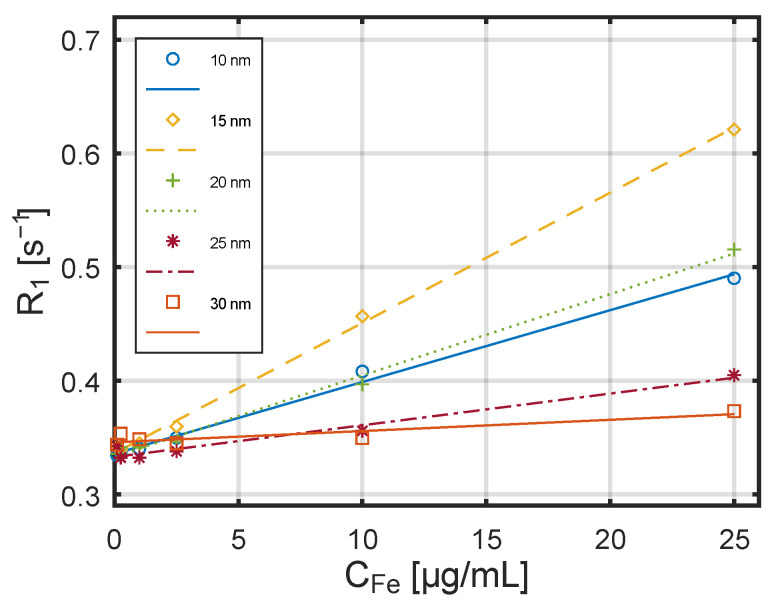
The dependance of the relaxation constant R_1_ on the concentration of SPA nanoparticles obtained by a Bruker scanner in a 7 T magnetic field.

The dependence of the relaxation constant R_1_ on the concentration of SPA nanoparticles obtained by the Bruker scanner in a 7 T magnetic field is shown in Figure 10.

**Figure 11:**
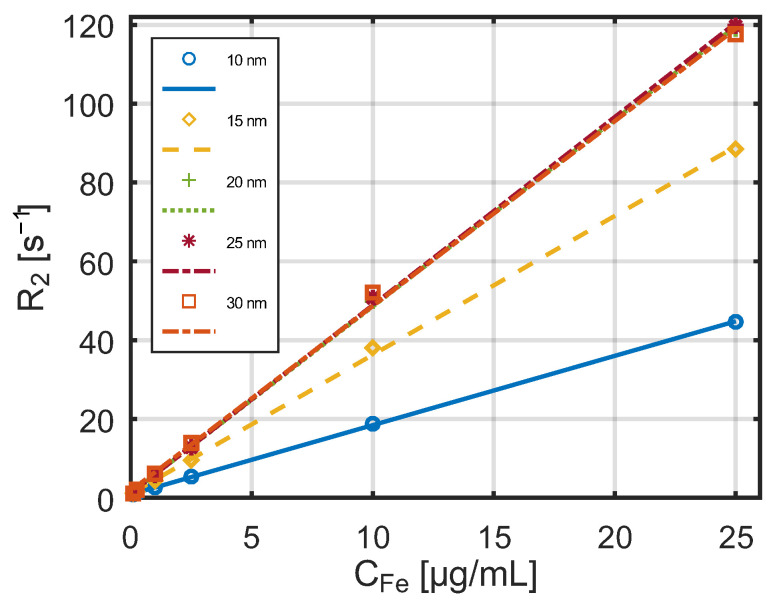
Dependence of the relaxation constant R_2_ on the concentration of SPA nanoparticles measured by a Bruker scanner in a 7 T magnetic field.

Also for the stronger magnetic field, a linear relationship is visible between the reciprocals of the relaxation times and the nanoparticle concentration. The dependence of the relaxation constant R_2_ on the concentration of these nanoparticles at the same magnetic field strength is shown in Figure 11. Relaxation constants vary approximately linearly with the concentration of nanoparticles, which is consistent with other studies [44]. An important parameter describing the efficiency of a contrast agent is its relaxivities r_1_ and r_2_, defined as the relaxation rates R_1_ and R_2_, respectively, normalized by the iron concentration. These coefficients can be interpreted as the increase in relaxation rate per unit concentration of nanoparticles (calculated as iron concentration).

**Figure 12:**
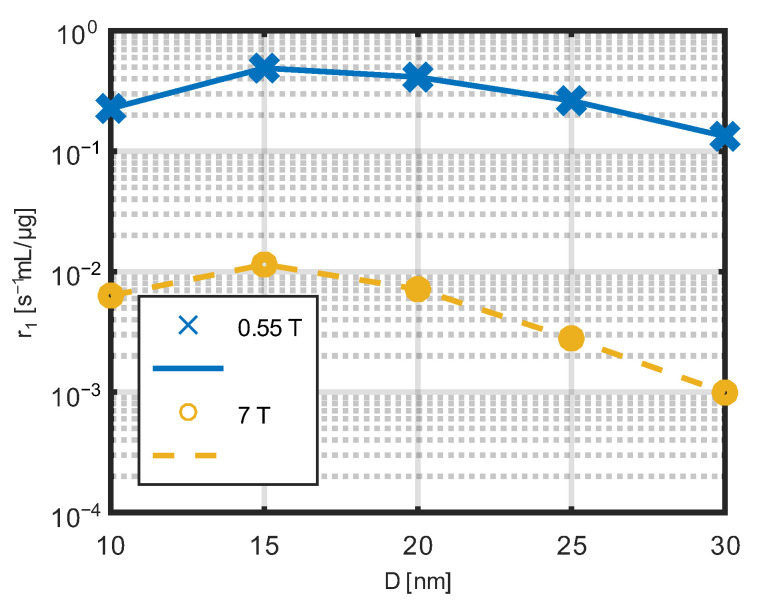
Dependence of the derivative r_1_ of the relaxation constant R_1_ with respect to the nanoparticle diameter for two magnetic fields.

The dependence of the relaxivity r_1_ with respect to the nanoparticle diameter for low field 0.55 T and high field 7T is presented in Figure 12. The maximum is visible for nanoparticles with a diameter of 15 nm for both field strengths.

**Figure 13:**
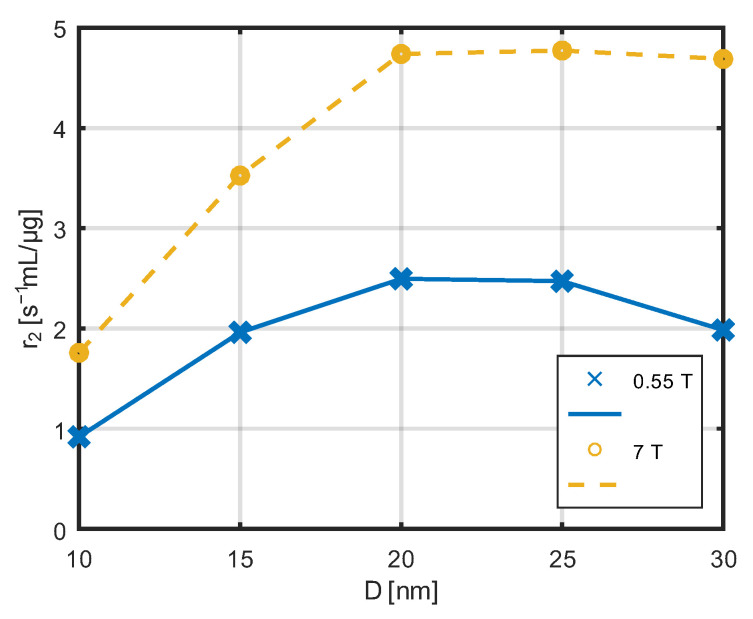
Dependence of the derivative r_2_ of the relaxation constant R_2_ with respect to the nanoparticle diameter for two magnetic fields.

The dependence of the relaxivity r_2_ with respect to the diameter of the nanoparticles for low and high field strength is presented in Figure 13. The maximum is visible for nanoparticles with a diameter of about 20–25 nm.

The observed significant decrease in longitudinal relaxivity (r_1_) at 7 T compared to lower field strengths (e.g., 0.55 T) is consistent with previous reports on the field dependence of relaxivity for paramagnetic and magnetic nanoparticle contrast agents [49,56]. It is well known that r_1_ relaxivity tends to decrease at high magnetic fields, while transverse relaxivity (r_2_) often increases or is less affected by field strength.

For example, Gd-based nanoparticles showed a reduction in r_1_ between lower and high fields, with r_1_ values at 7 T and 9.4 T significantly lower than at 3 T [56].

### 3.4. Measurements of Nanoparticle Heating Power and Relaxation Properties 

**Figure 14:**
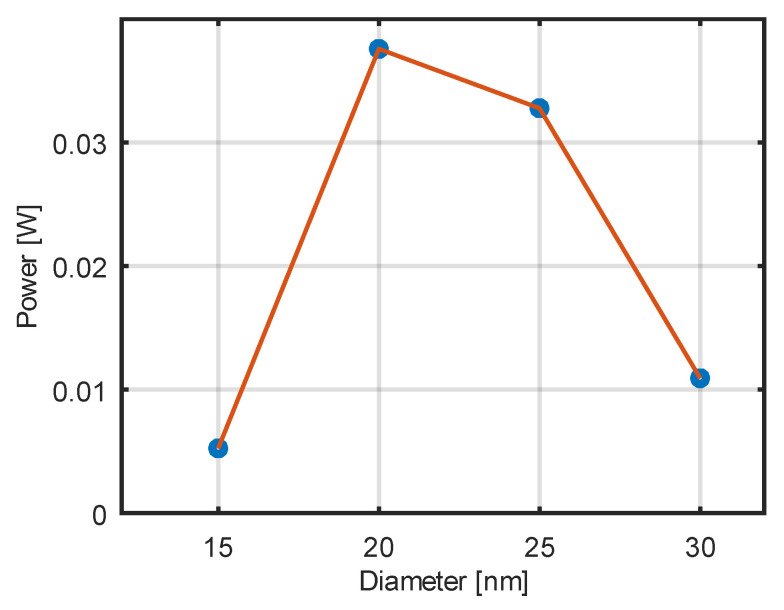
Dependence of the magnetic power losses of nanoparticles on core diameter at frequency 58.3 kHz. The blue dots in the plot indicate the results of the measurements.

A series of studies on the influence of nanoparticles with different diameters on calorimetric measurements of the heating power of nanoparticle suspensions was conducted. The temperature increases of nanoparticles were measured over time when exposed to an alternating magnetic field with a frequency of 58.3 kHz and an induction of 20 mT. The concentration of nanoparticles was 25 mg/mL. The duration of time during which the nanoparticles were exposed to an alternating magnetic field was 3600 s. The power loss values for nanoparticles of different diameters are shown in Figure 14. A maximum in the heating efficiency of the nanoparticles is observed for diameters in the range of 20–25 nm, which corresponds to the diameter range where the r_2_ relaxivity reaches its maximum, thereby maximizing the nanoparticles’ heating efficiency.

Table 1 presents the correlation coefficients between the measurements of relaxation times T_1_ and T_2_ taken in magnetic fields of 0.55 T and 7 T, as well as the measurements of heating power. Statistically significant correlations were identified for *p*-values lower than 0.05, while correlations with 0.05 ≤ *p* < 0.1 were treated as trends and are indicated accordingly.

Where the calculated correlation coefficient is statistically significant, the corresponding *p*-value is provided. From Table 1, it can be seen that statistically significant correlations exist between the T_1_ measurements in low (0.55 T) and high (7 T) magnetic fields, the T_2_ measurements in low and high fields, and between the heating power measurements and the T_2_ relaxation time measurements in the low field. The conducted measurements confirmed that one of the key parameters of magnetic nanoparticles is their diameter. The diameter of the nanoparticles affects both the relaxation times in low and high static magnetic fields, as well as the heating efficiency of the nanoparticles in an alternating magnetic field. For hyperthermia applications, the diameter range that ensures the highest heating efficiency should be selected. Additionally, for use as contrast agents, in order to minimize their concentration in tissues, the diameter range corresponding to the highest relaxivity values should be chosen.

The statistically significant correlations observed between T1 measurements at low (0.55 T) and high (7 T) magnetic fields, as well as between T2 measurements at both fields, indicate that despite the strong field dependence of absolute relaxivity values, the relative trends among the investigated nanoparticle samples remain preserved. This suggests that intrinsic nanoparticle properties, such as size distribution, magnetic anisotropy, and core magnetization, consistently influence relaxation processes across different magnetic field strengths. The strong correlation between T2 relaxation times measured at low and high fields is expected, as transverse relaxation in magnetic nanoparticles is predominantly governed by static and dynamic local field inhomogeneities, which scale similarly with particle magnetization at both field strengths.

Furthermore, the statistically significant correlation between the heating power and T2 relaxation time measured at low magnetic fields reflects the common physical origin of both phenomena, namely, magnetic losses associated with Néel and Brownian relaxation mechanisms. Particles exhibiting stronger transverse relaxation effects also tend to dissipate more energy in an alternating magnetic field, leading to higher heating power.

## 4. Discussion

The discussion of the obtained results is structured as follows. First, the structural characterization confirmed that the investigated nanoparticle samples were well defined in terms of core size and hydrodynamic diameter. The good agreement between TEM, STEM, and DLS measurements indicates a narrow size distribution and validates the reliability of further relaxometric and calorimetric analyses.

Second, the influence of nanoparticle size on longitudinal and transverse relaxivities was analyzed. The observed maximum of r_1_ relaxivity for nanoparticles with a core diameter of approximately 15 nm can be attributed to the balance between the magnetic moment magnitude and dynamic averaging of local magnetic field fluctuations. For smaller particles, thermal fluctuations reduce the effective magnetic moment, while for larger particles, slower magnetic dynamics limit the efficiency of T_1_ relaxation enhancement.

In contrast, r_2_ relaxivity reaches its maximum for larger nanoparticles with diameters in the range of 20–25 nm. Transverse relaxation is primarily governed by static and slowly varying magnetic field inhomogeneities induced by the nanoparticle magnetic moments. As the particle size and magnetic moment increase, these inhomogeneities become stronger, leading to enhanced dephasing of proton spins and increased r_2_ relaxivity.

Third, a strong dependence of r_1_ relaxivity on the applied magnetic field was observed. At 7 T, the nanoparticles exhibited a negligible effect on the T_1_ relaxation time within the investigated concentration range. This behavior is consistent with the well-known reduction in longitudinal relaxivity at high magnetic fields, where the Larmor frequency exceeds the characteristic fluctuation frequencies of the nanoparticle magnetic moments.

Finally, the calorimetric measurements revealed that the maximum heating power occurs for nanoparticles with diameters between 20 and 25 nm, which coincides with the size range where r_2_ relaxivity is maximized. This correlation reflects the common physical origin of both phenomena, namely, magnetic losses associated with Néel and Brownian relaxation processes in single-domain nanoparticles. As both heating efficiency and transverse relaxivity depend on the magnetic moment of the nanoparticles, an optimal particle size exists that allows efficient application in both magnetic hyperthermia and MRI contrast enhancement.

These results demonstrate the potential of the investigated nanoparticles for theranostic applications, where MRI can be used to visualize nanoparticle distribution prior to hyperthermia treatment induced by an alternating magnetic field.

## 5. Conclusions

This study demonstrates that the magnetic, relaxometric, and heating properties of iron oxide nanoparticles are governed by a common physical parameter, namely, the magnetic moment associated with particle size. An optimal nanoparticle diameter range of 20–25 nm was identified, for which both the transverse relaxivity (r_2_) and the heating efficiency in an alternating magnetic field reach their maximum values.

The observed overlap between the size ranges maximizing r_2_ relaxivity and heating power indicates that transverse spin dephasing and magnetic energy dissipation originate from the same relaxation mechanisms in single-domain nanoparticles. This finding provides a physical explanation for the strong correlation between r_2_ relaxivity and hyperthermia efficiency observed experimentally.

As a result, the same nanoparticle formulation can be effectively used for T_2_-weighted MRI contrast enhancement and magnetic hyperthermia therapy. This enables a theranostic approach in which MRI can be employed to assess nanoparticle distribution in tissue prior to the application of an alternating magnetic field for localized thermal treatment.

## Figures and Tables

**Table 1:** The correlation coefficients between the measurements of various parameters. All correlation coefficients are dimensionless.

	T1 (0.55 T)	T2 (0.55 T)	T1 (7 T)	T2 (7 T)	Power
T1 (0.55 T)	1	0.307	0.896 (*p* < 0.05)	0.021	0.457
T2 (0.55 T)	0.307	1	−0.141	0.943 (*p* < 0.05)	0.830 (*p* < 0.1)
T1 (7 T)	0.896 (*p* < 0.05)	−0.141	1	−0.401	0.057
T2 (7 T)	0.021	0.943 (*p* < 0.05)	−0.401	1	0.643
Power	0.457	0.830 (*p* < 0.1)	0.057	0.643	1

## Data Availability

The original contributions presented in this study are included in the article. Further inquiries can be directed to the corresponding author.

## Abbreviations

The following abbreviations are used in this manuscript:

NMR	Nuclear Magnetic Resonance
DLS	Dynamic Light Scattering
MRI	Magnetic Resonance Imaging
MAR	Motional Average Regime

## References

[1] Stueber D.D., Villanova J., Aponte I., Xiao Z., Colvin V.L. (2021). Magnetic Nanoparticles in Biology and Medicine: Past, Present, and Future Trends. Pharmaceutics.

[2] Usov N.A., Liubimov B.Y. (2012). Dynamics of magnetic nanoparticle in a viscous liquid: Application to magnetic nanoparticle hyperthermia. J. Appl. Phys..

[3] Martínez-Banderas A.I., Aires A., Plaza-García S., Colás L., Moreno J.A., Ravasi T., Merzaban J.S., Ramos-Cabrer P., Cortajarena A.L., Kosel J. (2020). Magnetic core–shell nanowires as MRI contrast agents for cell tracking. J. Nanobiotechnol..

[4] Singh V., Banerjee V. (2012). Ferromagnetism, hysteresis and enhanced heat dissipation in assemblies of superparamagnetic nanoparticles. J. Appl. Phys..

[5] Pankhurst Q.A., Thanh N.T.K., Jones S.K., Dobson J. (2009). Progress in applications of magnetic nanoparticles in biomedicine. J. Phys. D Appl. Phys..

[6] Gossuin Y., Gillis P., Lo Bue F. (2002). Susceptibility-induced T2-shortening and unrestricted diffusion. Magn. Reson. Med..

[7] Hobson N.J., Weng X., Siow B., Veiga C., Ashford M., Thanh N.T., Schätzlein A.G., Uchegbu I.F. (2019). Clustering superparamagnetic iron oxide nanoparticles produces organ-targeted high-contrast magnetic resonance images. Nanomedicine.

[8] Manescu V., Antoniac I., Paltanea G., Nemoianu I.V., Mohan A.G., Antoniac A., Rau J.V., Laptoiu S.A., Mihai P., Gavrila H. (2024). Magnetic Hyperthermia in Glioblastoma Multiforme Treatment. Int. J. Mol. Sci..

[9] Rentzeperis F., Rivera D., Zhang J.Y., Brown C., Young T., Rodriguez B., Schupper A., Price G., Gomberg J., Williams T. (2024). Recent Developments in Magnetic Hyperthermia Therapy (MHT) and Magnetic Particle Imaging (MPI) in the Brain Tumor Field: A Scoping Review and Meta-Analysis. Micromachines.

[10] Gomes A.A., Valverde T.M., Machado V.d.O., do Nascimento da Silva E., Fagundes D.A., Oliveira F.d.P., Freitas E.T.F., Ardisson J.D., Ferreira J.M.d.F., Oliveira J.A.d.C. (2024). Heating Capacity and Biocompatibility of Hybrid Nanoparticles for Magnetic Hyperthermia Treatment. Int. J. Mol. Sci..

[11] Yadel C., Michel A., Casale S., Fresnais J. (2018). Hyperthermia Efficiency of Magnetic Nanoparticles in Dense Aggregates of Cerium Oxide/Iron Oxide Nanoparticles. Appl. Sci..

[12] Obaidat I.M., Narayanaswamy V., Alaabed S., Sambasivam S., Muralee Gopi C.V.V. (2019). Principles of Magnetic Hyperthermia: A Focus on Using Multifunctional Hybrid Magnetic Nanoparticles. Magnetochemistry.

[13] Spirou S.V., Basini M., Lascialfari A., Sangregorio C., Innocenti C. (2018). Magnetic Hyperthermia and Radiation Therapy: Radiobiological Principles and Current Practice. Nanomaterials.

[14] Vilas-Boas V., Carvalho F., Espiña B. (2020). Magnetic Hyperthermia for Cancer Treatment: Main Parameters Affecting the Outcome of In Vitro and In Vivo Studies. Molecules.

[15] Szwed M., Marczak A. (2024). Application of Nanoparticles for Magnetic Hyperthermia for Cancer Treatment—The Current State of Knowledge. Cancers.

[16] Tsampasian V., Merinopoulos I., Cameron D., Garg P., Vassiliou V.S. (2022). Ultrasmall Superparamagnetic Particles of Iron Oxide and Cardiac Magnetic Resonance: Novel Imaging in Everyday Conditions. Appl. Sci..

[17] Iacob N. (2025). Pitfalls and Challenges in Specific Absorption Rate Evaluation for Functionalized and Coated Magnetic Nanoparticles Used in Magnetic Fluid Hyperthermia. Coatings.

[18] Kerroum M.A.A., Iacovita C., Baaziz W., Ihiawakrim D., Rogez G., Benaissa M., Lucaciu C.M., Ersen O. (2020). Quantitative Analysis of the Specific Absorption Rate Dependence on the Magnetic Field Strength in Zn_x_Fe_3−x_O_4_ Nanoparticles. Int. J. Mol. Sci..

[19] Dias A.M.M., Courteau A., Bellaye P.-S., Kohli E., Oudot A., Doulain P.-E., Petitot C., Walker P.-M., Decréau R., Collin B. (2022). Superparamagnetic Iron Oxide Nanoparticles for Immunotherapy of Cancers through Macrophages and Magnetic Hyperthermia. Pharmaceutics.

[20] Issa B. (2018). Reduction of T2 relaxation rates due to large volume fractions of magnetic nanoparticles for all motional regimes. Appl. Sci..

[21] Zhao Z., Li M., Zeng J., Huo L., Liu K., Wei R., Ni K., Gao J. (2022). Recent advances in engineering iron oxide nanoparticles for effective magnetic resonance imaging. Bioact. Mater..

[22] Ruiz-González M.L., Torres-Pardo A., González-Calbet J.M. (2021). The Role of Transmission Electron Microscopy in the Early Development of Mesoporous Materials for Tissue Regeneration and Drug Delivery Applications. Pharmaceutics.

[23] Malatesta M. (2022). Transmission Electron Microscopy as a Powerful Tool to Investigate the Interaction of Nanoparticles with Subcellular Structures. Int. J. Mol. Sci..

[24] Rodriguez-Loya J., Lerma M., Gardea-Torresdey J.L. (2024). Dynamic Light Scattering and Its Application to Control Nanoparticle Aggregation in Colloidal Systems: A Review. Micromachines.

[25] Anzini P., Redoglio D., Rocco M., Masciocchi N., Ferri F. (2022). Light Scattering and Turbidimetry Techniques for the Charac-terization of Nanoparticles and Nanostructured Networks. Nanomaterials.

[26] Zhang D., Zhang J., Bian X., Zhang P., Wu W., Zuo X. (2025). Iron Oxide Nanoparticle-BasedT1 Contrast Agents for Magnetic Resonance Imaging: A Review. Nanomaterials.

[27] Geraldes C.F.G.C. (2024). Rational Design of Magnetic Nanoparticles as T1–T2 Dual-Mode MRI Contrast Agents. Molecules.

[28] Delangre S., Vuong Q., Henrard D., Magat J., Po C., Gallez B., Gossuin Y. (2015). Theoretical and experimental study of ON-resonance saturation, an MRI sequence for positive contrast with superparamagnetic nanoparticles. J. Magn. Reson..

[29] Nowak-Jary J., Machnicka B. (2024). Comprehensive Analysis of the Potential Toxicity of Magnetic Iron Oxide Nanoparticles for Medical Applications: Cellular Mechanisms and Systemic Effects. Int. J. Mol. Sci..

[30] Boyadzhiev A., Wu D., Avramescu M.-L., Williams A., Rasmussen P., Halappanavar S. (2024). Toxicity of Metal Oxide Nanoparticles: Looking through the Lens of Toxicogenomics. Int. J. Mol. Sci..

[31] Malhotra N., Lee J.-S., Liman R.A.D., Ruallo J.M.S., Villaflores O.B., Ger T.-R., Hsiao C.-D. (2020). Potential Toxicity of Iron Oxide Magnetic Nanoparticles: A Review. Molecules.

[32] Nowak-Jary J., Machnicka B. (2025). Toxicity of Magnetic Nanoparticles in Medicine: Contributing Factors and Modern Assessment Methods. Int. J. Mol. Sci..

[33] Delangre S., Vuong Q.L., Po C., Gallez B., Gossuin Y. (2016). Improvement of the Off-Resonance Saturation, an MRI sequence for positive contrast with SPM particles: Theoretical and experimental study. J. Magn. Reson..

[34] Midura M., Wróblewski P., Wanta D., Kryszyn J., Smolik W.T., Domański G., Wieteska M., Obrębski W., Piątkowska-Janko E., Bogorodzki P. (2022). The Hybrid System for the Magnetic Characterization of Superparamagnetic Nanoparticles. Sensors.

[35] Harabech M., Leliaerta J., Coene A., Crevecoeur G., Van Roost D., Dupré L. (2017). The effect of the magnetic nanoparticle’s size dependence of the relaxation time constant on the specific loss power of magnetic nanoparticle hyperthermia. J. Magn. Magn. Mater..

[36] Deatsch A.E., Evans B. (2014). Heating efficiency in magnetic nanoparticle hyperthermia. J. Magn. Magn. Mater..

[37] Hergt R., Dutz S., Müller R., Zeisberger M. (2006). Magnetic particle hyperthermia: Nanoparticle magnetism and materials development for cancer therapy. J. Phys. Condens. Matter.

[38] Du Y., Lai P.T., Leung C.H., Pong P.W.T. (2013). Design of Superparamagnetic Nanoparticles for Magnetic Particle Imaging (MPI). Nanoparticles. Int. J. Mol. Sci..

[39] Caizer C. (2021). Optimization Study on Specific Loss Power in Superparamagnetic Hyperthermia with Magnetite Nanoparticles for High Efficiency in Alternative Cancer Therapy. Nanomaterials.

[40] Rosensweig R.E. (2002). Heating magnetic fluid with alternating magnetic field. J. Magn. Magn. Mater..

[41] Maniotis N., Maragakis M., Vordos N. (2025). A comprehensive analysis of nanomagnetism models for the evaluation of particle energy in magnetic hyperthermia. Nanoscale Adv..

[42] Vallejo-Fernandez G., Whear O., Roca A.G., Hussain S., Timmis J., Patel V., O’Grady K. (2013). Mechanisms of hyperthermia in magnetic nanoparticles. J. Phys. D Appl. Phys..

[43] Pankhurst Q.A., Connolly J., Jones S.K., Dobson J. (2003). Applications of magnetic nanoparticles in biomedicine. J. Phys. D Appl. Phys..

[44] Weisskoff R.M., Zuo C.S., Boxerman J.L., Rosen B.R. (1994). Microscopic susceptibility variation and transverse relaxation: Theory and experiment. Magn. Reson. Med..

[45] Brown K.A., Vassiliou C.C., Issadore D., Berezovsky J., Cima M.J., Westervelt R.M. (2010). Scaling of transverse nuclear magnetic relaxation due to magnetic nanoparticle aggregation. J. Magn. Magn. Mater..

[46] Jensen J.H., Chandra R. (2000). NMR relaxation in tissues with weak magnetic inhomogeneities. Magn. Reson. Med..

[47] Gillis P., Francis M., Rodney A.B. (2002). On T2-shortening by strongly magnetized spheres: A partial refocusing model. Magn. Reson. Med..

[48] Kostevšek N. (2020). A Review on the Optimal Design of Magnetic Nanoparticle-Based T2 MRI Contrast Agents. Magnetochemistry.

[49] Reale G., Calderoni F., Ghirardi T., Porto F., Illuminati F., Marvelli L., Martini P., Uccelli L., Tonini E., Del Bianco L. (2023). Development and Evaluation of the Magnetic Properties of a New Manganese (II) Complex: A Potential MRI Contrast Agent. Int. J. Mol. Sci..

[50] Roch A., Muller R.N., Gillis P. (1999). Theory of proton relaxation induced by superparamagnetic particles. J. Chem. Phys..

[51] Gillis P., Koening S.H. (1987). Transverse Relaxation of Solvent Protons Induced by Magnetized Spheres: Application to Ferritin, Erythrocytes, and Magnetite. Magn. Reson. Med..

[52] Ocean NanoTech Product Data Sheet. Iron Oxide Nanoparticles in Water. https://oceannanotech.com/web/upload/pdfs/specifications/SPA_Specification_Sheet.pdf.

[53] El-Gendy A.A., Ibrahim E.M.M., Khavrus V.O., Krupskaya Y., Hampel S., Leonhardt A., Buchner B., Klingeler R. (2009). The synthesis of carbon coated Fe, Co and Ni nanoparticles and an examination of their magnetic properties. Carbon.

[54] Otsu N. (1979). A Threshold Selection Method from Gray-Level Histograms. IEEE Trans. Syst. Man Cybern..

[55] Jia Z., Li J., Gao L., Yang D., Kanaev A. (2023). Dynamic Light Scattering: A Powerful Tool for In Situ Nanoparticle Sizing. Colloids Interfaces.

[56] Hagberg G.E., Scheffler K. (2013). Effect of r_1_ and r_2_ relaxivity of gadolinium-based contrast agents on the T_1_-weighted MR signal at increasing magnetic field strengths. Contrast Media Mol. Imaging.

